# Peripherally Acting Opioids in Orofacial Pain

**DOI:** 10.3389/fnins.2021.665445

**Published:** 2021-05-04

**Authors:** Qing Liu, Hongwen He, Lijia Mai, Shengyan Yang, Wenguo Fan, Fang Huang

**Affiliations:** ^1^Department of Pediatric Dentistry, Guanghua School of Stomatology, Hospital of Stomatology, Sun Yat-sen University, Guangzhou, China; ^2^Guangdong Provincial Key Laboratory of Stomatology, Guangzhou, China; ^3^Department of Oral Anatomy and Physiology, Guanghua School of Stomatology, Hospital of Stomatology, Sun Yat-sen University, Guangzhou, China

**Keywords:** peripheral opioid receptors, endogenous opioid peptides, orofacial pain, immune cells, neuroimmune

## Abstract

The activation of opioid receptors by exogenous or endogenous opioids can produce significant analgesic effects in peripheral tissues. Numerous researchers have demonstrated the expression of peripheral opioid receptors (PORs) and endogenous opioid peptides (EOPs) in the orofacial region. Growing evidence has shown the involvement of PORs and immune cell-derived EOPs in the modulation of orofacial pain. In this review, we discuss the role of PORs and EOPs in orofacial pain and the possible cellular mechanisms involved. Furthermore, the potential development of therapeutic strategies for orofacial pain is also summarized.

## Introduction

It has been well established that opioid receptors are widely distributed throughout the central (spinal cord, brain) and peripheral (trigeminal and dorsal root ganglia) sensory nervous systems (Machelska and Celik, 2020). Activation of opioid receptors by opioids can play a significant role in inflammatory and neuropathic pain. A growing body of literature has noted that interactions involving peripheral opioid receptors (PORs) and immune cell-derived endogenous opioid peptides (EOPs) produce potent analgesia in painful conditions (Machelska and Stein, 2006; Hua, 2016). Furthermore, the analgesic effects of PORs and EOPs occur without central side effects, such as respiratory depression, nausea, constipation, pruritus, addiction, and tolerance (Stein, 2016). PORs and EOPs have been shown to be involved in orofacial pain (Liu et al., 2020). This review provides evidence for the role of PORs and immune cell-derived EOPs in orofacial pain. Possible cellular mechanisms involving PORs and EOPs in modulating orofacial pain are also discussed. In addition, this review summarizes potential development of therapeutic strategies for orofacial pain control.

## The Opioid System

### Opioid Receptors

Opioid receptors are classified into three main subtypes: μ-receptor (MOR), δ-receptor (DOR) and κ-receptor (KOR) (Cox, 2013; Stevens, 2015). These three opioid receptors belong to the family of G-nucleotide binding protein-coupled receptors (GPCRs) and are encoded by three genes: *Oprm1, Oprd1*, and *Oprk1* (Law et al., 2013). The structure of opioid receptors consists of seven transmembrane domains, three intracellular hydrophobic loops, three extracellular loops, an extracellular amino terminus, and an intracellular carboxyl terminus (Sobczak et al., 2014).

Opioid receptors are synthesized in the dorsal root ganglion (DRG) and transported to central and peripheral nerve terminals. In the peripheral nervous system, opioid receptors are expressed in the cell bodies and peripheral terminals of primary afferent neurons and sympathetic postganglionic terminals (Stein, 2003; Sehgal et al., 2011). In addition to the nervous system, opioid receptors are also expressed by immune cells, such as lymphocytes, macrophages, and granulocytes (Chuang et al., 1995a,b; Celik et al., 2016; Jiao et al., 2018). MOR, DOR, and KOR proteins can be detected in astrocytes and oligodendrocytes in the mouse brain (Stiene-Martin et al., 2001), but no study has shown the expression of opioid receptors in satellite glial cells. More research is needed to verify the presence of opioid receptors in glial cells.

### Endogenous Opioid Peptides

Classical EOPs are categorized as endorphins, enkephalins, and dynorphins, which are derived from three precursor proteins, proopiomelanocortin (POMC), proenkephalin (PENK), and prodynorphin (PDYN), respectively (Stein, 2018). These opioid peptides exhibit different affinity and selectivity for MOR (endorphins, enkephalins), DOR (enkephalins, endorphins) and KOR (dynorphins) (Machelska, 2007). It has been demonstrated that EOPs are expressed in the central and peripheral nervous systems, neuroendocrine tissues, and immune cells (Labuz et al., 2009; Stein and Machelska, 2011; Basso et al., 2015; Table 1).

**TABLE 1:** Characterization of the opioid system.

Opioid receptors	Precursor proteins	Endogenous opioid peptides	Exogenous agonists	Exogenous antagonists	Effects	References
MOR	POMC	β-endorphin* Endomorphin-1 Endomorphin-2	DAMGO Morphine Fentanyl	Naloxone Naltrexone CTOP	Analgesia, respiratory depression, euphoria, constipation, nausea, dependence	Chan et al., 2017; Zhu et al., 2018; Grim et al., 2020
DOR	PENK	[Met^5^]Enkephalin* [Leu^5^]Enkephalin	DELTs DPDPE SNC80	Naltrindole Naloxone ICI 174,864	Analgesia, convulsions, reward	Jutkiewicz et al., 2005; Vicente-Sanchez et al., 2016; Dripps et al., 2020
KOR	PDYN	Dynorphin A Dynorphin B α-neomorphin β-neomorphin	U-69,593 U50,488 Bremazocine	Naloxone Naltrexone NorBNI	Analgesia, diuresis, dysphoria, sedation,	Brust et al., 2016; Helal et al., 2017

*^∗^β-endorphin also binds DOR; Enkephalins also bind MOR. MOR, μ-opioid receptor; DOR, δ-opioid receptor; KOR, κ-opioid receptor; POMC, proopiomelanocortin; PENK, proenkephalin; PDYN, prodynorphin; DAMGO, [D-Ala2,N-Me-Phe4,Gly5-ol]-enkephalin; DPDPE, D-Pen2, D-Pen5-enkpephalin; SNC80, 4-(alpha-(4-allyl-2,5-dimethyl-1-piperazinyl)-3-methoxybenzyl)-N,N-diethylbenzamide; U-69,593, (+)-(5α,7α,8β)-N-Methyl-N-[7-(1-pyrrolidinyl)-1-oxaspiro[4.5]dec-8-yl]-benzeneacetamide; U50,488, *trans-*(±)3,4-dichloro-N-methyl-N-[2-(1-pyrrolidinyl)-cyclohexyl]-benzeneacetamide; CTOP, D-Phe-Cys-Tyr-D-Trp-Orn-Thr-Pen-Thr-NH2; ICI 174,864, N, N-diallyl-Tyr-Aib-Aib-Phe-Leu; NorBNI, norbinaltorphimine.*

Moreover, EOPs or their precursors are also found in glial cells. Several studies have identified the expression of opioid peptides in cultured primary spinal microglia and astrocytes (Vilijn et al., 1988; Shinoda et al., 1989; Hauser et al., 1990). Spinal cord microglia-derived β-endorphins have been shown to participate in inflammatory, neuropathic, and bone cancer pain (Gong et al., 2014; Fan et al., 2016; Huang et al., 2017; Wu et al., 2018; Mao et al., 2019). However, there are no studies reported concerning the expression of opioid peptides in satellite glial cells.

Opioid-releasing immune cells mainly include macrophages/monocytes, granulocytes, and lymphocytes (Mousa et al., 2004, 2007; Pannell et al., 2016). The expression of EOPs and their precursor proteins in immune cells is increased during inflammation (Cabot et al., 1997; Boue et al., 2011; Hua, 2016). In the initial phase of inflammation, the major opioid-producing leukocytes are granulocytes, while macrophages/monocytes and lymphocytes predominate at later stages of inflammation (Rittner et al., 2001; Brack et al., 2004a). IL-4 can upregulate POMC expression and β-endorphin production in lymphocytes, which contributes to the inhibition of inflammatory pain via activation of PORs (Busch-Dienstfertig et al., 2012). Enkephalins released by CD4(+) T lymphocytes relieve visceral inflammatory pain (Basso et al., 2018). Another study indicates that increased levels of methionine-enkephalin (Met-enkephalin) and dynorphin A produce analgesic effects against long-lasting inflammatory pain induced by complete Freund’s adjuvant (CFA) (Jiang et al., 2015). The analgesic effects of immune cell-derived EOPs have also been verified for neuropathic and cancer pain (Baamonde et al., 2006; Labuz et al., 2009, 2010).

## Modulation of Orofacial Pain via PORS and EOPS

### Orofacial Pain

Orofacial pain is the common name of various disorders, ranging from inflammatory diseases to neuropathic pain syndromes, and which refers to pain associated with the head, face, neck, and intraoral structures (Ayoub et al., 2018; Feher et al., 2019). The prevalence of orofacial pain is approximately 17 to 26%, of which 7–11% is classified as chronic (Benoliel and Sharav, 2010). Inflammatory mediators produced during orofacial inflammation and peripheral nerve damage or infection can evoke pain via activation and sensitization of specialized trigeminal primary afferent neurons called nociceptors (Rittner et al., 2008; Matsuda et al., 2019). Nociceptors mainly comprise myelinated Aδ and non-myelinated C fibers, and their cell bodies are located in the trigeminal ganglion (TG) (Sessle, 2011). After activation by noxious stimuli, these nociceptive fibers conduct nerve impulses from their nociceptive endings into the somatosensory and limbic cortices through the spinal trigeminal nucleus and thalamus.

Upon peripheral inflammation or nerve injury, numerous non-neuronal cells, such as endothelial cells, fibroblasts, dendritic cells, mast cells, macrophages, and Schwann cells are activated and release many mediators (Machelska, 2011). The inflammatory mediators involved in the activation of nociceptors include, but are not limited to, cytokines, chemokines, nerve growth factor, bradykinin, prostaglandins, and ATP (Rittner and Stein, 2005). Activation of various ion channels and protein kinases, such as transient receptor potential vanilloid 1 (TRPV1), protein kinase A, and mitogen-activated protein kinases (MAPKs), also contribute to the development of pain and hyperalgesia (Aley and Levine, 1999; Obata and Noguchi, 2004; Moore et al., 2018). A better understanding of the underlying orofacial inflammatory and neuropathic pain mechanism will allow the development of novel therapeutic strategies for such painful conditions.

### The Role of PORs and EOPs in Orofacial Pain

Accumulating evidence has shown that PORs are expressed in orofacial tissue. An early study indicated the immunohistochemical localization of DOR in various peripheral tissues, including the cornea, eyelid, and lip (Wenk and Honda, 1999). A number of investigations have demonstrated increased expression of MOR protein via western blot and immunofluorescent staining in TG under acute muscle pain conditions (Bagues et al., 2018; Zhang et al., 2018). The upregulation of MOR in TG is observed in rat orofacial inflammatory pain models utilizing RT-PCR at the mRNA level and western blot at the protein level (Nunez et al., 2007; Zhang et al., 2014). The levels of MOR mRNA detected by qPCR and of protein detected by immunohistochemistry and western blot are elevated in the rat orofacial persistent pain model (Bai et al., 2015). Immunohistochemical experiments and qPCR revealed that the androgen receptor regulates MOR expression in the TG under acute masseter pain conditions (Lee et al., 2016). The level of MOR in the anterior synovial membrane is higher than that in the posterior synovial membrane in the non-inflamed temporomandibular joint (TMJ) using non-radiographic *in situ* hybridization and immunohistochemistry (Hayashi et al., 2002). Moreover, the application of peripherally acting KOR agonists suppresses formalin-induced TMJ nociceptive behavior, indicating the location of functional KOR within the rat TMJ (Clemente et al., 2004). Several immunohistochemical studies have revealed the localization of MOR and DOR in human and rat dental pulp (Jaber et al., 2003; Fristad et al., 2006). Furthermore, the expression of MOR, DOR, and KOR has been verified in primary human oral epithelial cells by RT-PCR and immunocytochemical techniques (Charbaji et al., 2012; Table 2).

**TABLE 2:** The expression of EOPs and PORs in orofacial tissues.

Opioid receptor/opioid peptide	Location of expression	Species	mRNA/protein	Methods	References
MOR	TG	Mouse	Protein	Immunofluorescence staining	Zhang et al., 2018
	TG	Rat	Protein	Western Blot	Bagues et al., 2018
	TG	Rat	mRNA and protein	PCR, immunocytochemistry and Western Blot	Nunez et al., 2007; Zhang et al., 2014; Bai et al., 2015; Lee et al., 2016
	TMJ synovial membrane	Rat	mRNA and protein	*In situ* hybridization and immunohistochemistry	Hayashi et al., 2002
	Dental pulp	Human	Protein	Immunohistochemistry	Jaber et al., 2003
	Oral epithelial cells	Human	mRNA and protein	RT-PCR and immunocytochemistry	Charbaji et al., 2012
DOR	Corneas, eyelids, and the lip	Rat and macaque monkey	Protein	Immunohistochemistry	Wenk and Honda, 1999
	Dental pulp	Rat	Protein	Immunohistochemical and ultrastructural analysis	Fristad et al., 2006
	Oral epithelial cells	Human	mRNA and protein	RT-PCR and immunocytochemistry	Charbaji et al., 2012
	TG	Rat	mRNA	qPCR	Saloman et al., 2011
KOR	Oral epithelial cells	Human	mRNA and protein	RT-PCR and immunocytochemistry	Charbaji et al., 2012
Enkephalin	Dental pulp tissue	Rat	Precursor protein	Chromatography	Wei et al., 1991
	Dental pulp	Human	Protein	Radioimmunoassay	Chavarría-Bolaños et al., 2014; Chavarria-Bolanos et al., 2015
Endorphin	TG and the periodontium	Rat	Protein	Immunostaining assay	Liu et al., 2019
	Dental pulp	Human	Protein	Radioimmunoassay	Chavarría-Bolaños et al., 2014; Chavarria-Bolanos et al., 2015

Notably, activation of PORs can produce antinociceptive effects on orofacial inflammatory and neuropathic pain. The contribution of PORs to antinociception has been demonstrated in orofacial masseter pain (Sanchez et al., 2010; Saloman et al., 2011). The antinociceptive effects mediated by PORs were also assessed in rat TMJ pain models (Macedo et al., 2016; Coura et al., 2017). Activation of local opioid receptors by enkephalin attenuates nociceptive behavior in a rat model of trigeminal neuropathic pain (Meunier et al., 2005). Low doses of naloxone have been shown to produce peripheral antinociceptive effects by activating DOR and KOR in *in vivo* and *in vitro* models of trigeminal nociception (Capuano et al., 2010). Furthermore, therapeutic ultrasound is effective in alleviating experimental trigeminal neuropathic pain, with a mechanism involving opioid receptor activation (Savernini et al., 2012). Clinical studies have also assessed the efficacy of peripheral morphine analgesia in dental surgery-induced pain, oral mucositis pain, and temporomandibular disorder with myofascial pain (Likar et al., 2001; Nielsen et al., 2012; Kang et al., 2018).

Meanwhile, various investigations have indicated the involvement of EOPs in orofacial pain. An early chromatography study suggested the distribution of enkephalin precursor proteins in the nuclear, microsomal, and supernatant fractions of rat dental pulp tissue (Wei et al., 1991). Another immunostaining assay has shown that endomorphin-2 is basally expressed in TG, trigeminal nucleus caudalis, and periodontium in rats (Liu et al., 2019). Moreover, clinical studies have revealed increased expression levels of β-endorphins and Met-enkephalin in human dental pulp with asymptomatic inflammation or controlled orthodontic intrusive forces by radioimmunoassay (Chavarría-Bolaños et al., 2014, 2015). The administration of endomorphin-2 to both TG and periodontal tissues alleviates orofacial pain induced by tooth movement (Liu et al., 2019). Local overproduction of PENK-derived peptides in TG sensory neurons via a genomic herpes simplex virus-derived vector evokes a potent antiallodynic effect on trigeminal neuropathic pain by activating PORs (Meunier et al., 2005). In addition, resveratrol has been shown to inhibit the nociceptive jaw-opening reflex via the endogenous opioid system (Kokuba et al., 2017). Clinical trials have also demonstrated that β-endorphin levels are correlated with TMJ pain (Feldreich et al., 2012, 2017). Notably, β-endorphin and dynorphin released from leukocytes cells are involved in the antinociceptive effect of 15-deoxy-Δ12,14-prostaglandin J2 (15d-PGJ2) in formalin-induced TMJ pain (Macedo et al., 2016). Neutrophil-mediated β-endorphin also produces antinociceptive effects on acute oral cancer pain in rats (Scheff et al., 2018, 2019; Table 3). To date, few studies have focused on the EOPs released by immune cells in orofacial pain, which deserves further investigation.

**TABLE 3:** The role of EOPs and PORs in orofacial pain modulation.

Opioid receptor/opioid peptide	Orofacial pain model	Modeling approaches	Site of action	Effects	References
MOR	Orofacial muscle pain	HS	TG	Antinociceptive effect	Bagues et al., 2018
	Inflammatory orofacial muscle pain	CFA, HS	TG	Antinociception	Nunez et al., 2007
	Orofacial persistent pain	TASM ligation	TG	Anti-allodynia	Bai et al., 2015
	Orofacial myositis	CFA	TG	Anti-hyperalgesia	Zhang et al., 2014
	Masseter muscle pain	HS	Masseter	Antinociceptive effect	Sanchez et al., 2010
	TMJ pain	Formalin	TMJ	Antinociceptive effect	Coura et al., 2017
DOR	Acute orofacial muscle pain	Capsaicin	Masseter muscle	Anti-hyperalgesia	Saloman et al., 2011
	TMJ pain	Formalin	TMJ	Antinociceptive effect	Macedo et al., 2016; Coura et al., 2017
	Orofacial formalin test	Formalin	Trigeminal neurons	Antinociceptive effect	Capuano et al., 2010
KOR	TMJ inflammatory pain	Formalin	TMJ	Antinociception	Clemente et al., 2004
	TMJ pain	Formalin	TMJ	Antinociceptive effect	Macedo et al., 2016; Coura et al., 2017
	Orofacial formalin test	Formalin	Trigeminal neurons	Antinociceptive effect	Capuano et al., 2010
Endorphin	TMJ pain	Formalin	TMJ	Antinociceptive effect	Macedo et al., 2016
	Orthodontic pain	Orthodontic tooth movement	TG and periodontal tissues	Antinociceptive effect	Liu et al., 2019
	Oral cancer pain	Injecting cancer cell line supernatant	Tongue	Antinociception	Scheff et al., 2018, 2019
Dynorphin	TMJ pain	Formalin	TMJ	Antinociceptive effect	Macedo et al., 2016
Enkephalin	Trigeminal neuropathic pain	Unilateral chronic constriction injury to infraorbital nerve	TG, infraorbital nerves	Antiallodynic effect	Meunier et al., 2005

*HS, hypertonic saline; TASM, tendon of anterior superficial part of rat masseter muscle; TG, trigeminal ganglion; TMJ, temporomandibular joint; CFA, complete Freund’s adjuvant.*

### Mechanisms of Action

#### Migration of Opioid Peptide-Containing Immune Cells to Inflamed/Injured Tissue

It has been confirmed that granulocyte colony-stimulating factor significantly increases neutrophil infiltration in mouse tongues with oral cancer (Scheff et al., 2019). Administration of 1% thioglycollate in the TMJ enhances the recruitment of opioid peptide-containing leukocytes, especially macrophages, in TMJ periarticular tissue (Macedo et al., 2016). The upregulation of macrophages and lymphocytes can be observed following trigeminal nerve injury (Moreau et al., 2017). The recruitment of immune cells from the circulation into sites of nerve damage or inflamed tissue is a multistep process involving various adhesion molecules located on immune cells and vascular endothelium (Machelska, 2011; Iwaszkiewicz et al., 2013). Initially, circulating immunocytes roll along the vascular endothelial cell wall, mediated predominantly by P- and E-selectins on endothelial cells and L-selectin on immunocytes (Machelska et al., 2004). Subsequently, immunocytes are activated by chemokines secreted by endothelial and inflammatory cells and which are present on the luminal surface of the vascular endothelium (Stein et al., 2003; Busch-Dienstfertig and Stein, 2010). The expression level and avidity of integrins are upregulated, which mediates adhesion between immunocytes and endothelial cells via intercellular adhesion molecule-1 (ICAM-1) (Machelska et al., 2002; Machelska, 2007). Finally, the immunocytes migrate through the endothelium, directed predominantly by platelet-endothelial cell adhesion molecule-1 (PECAM-1) (Ley et al., 2007).

Studies have found that P-selectin is upregulated in hamster oral mucositis models, as is ICAM-1 in inflammatory process involving dental pulp (Chang et al., 2019; Mafra et al., 2019). Upregulation of P-selectin and PECAM-1, and co-expression of L-selectin and β-endorphin, are observed in immunocytes within inflamed tissues (Mousa et al., 2000). Furthermore, the depletion of neutrophil-expressed β-endorphin and Met-enkephalin induced by antibodies results in nociceptive behavior in mouse oral cancer pain models (Scheff et al., 2018). Pretreatment with antagonists of selectins, ICAM-1, integrins, and chemokines leads to decreased number of opioid peptide-containing cells in inflamed tissues and weakens the peripheral analgesic effect (Brack et al., 2004b; Machelska, 2007; Stein and Machelska, 2011). Additionally, intrathecal morphine results in a reduction in the number of β-endorphin-containing cells within the inflamed tissues, and peripheral endogenous antinociceptive effects are also significantly decreased. These findings suggest the role of central mechanisms in modulating peripheral endogenous opioid analgesia (Schmitt et al., 2003). However, another study has indicated the direct binding involving lymphocytes and cultured sensory neurons via adhesion molecules such as ICAM-1 and neural cell adhesion molecule (NCAM) (Hua et al., 2006), may be necessary to release EOPs within the effective range of PORs to produce adequate analgesia (Hua, 2016). Currently, few investigations have focused on the migration of immune cells expressing EOPs into inflamed/injured tissue upon orofacial pain, which requires further intensive study.

#### Release of EOPs From Immune Cells

In peripheral inflamed tissues or damaged nerves, opioid-containing immune cells release EOPs and then migrate to local lymph nodes (Hua and Cabot, 2010). Various factors trigger the release of EOPs by immune cells. Inflammatory mediators, such as corticotropin-releasing factor (CRF) and interleukin-1β (IL-1β), can stimulate the secretion of EOPs by immunocytes (Schafer et al., 1994; Cabot et al., 2001). Dental pulp injuries significantly increase CRF receptor expression in a rat model, which is correlated with localized increases in leukocytes and β-endorphins (Rutz et al., 2007; Uhrich et al., 2015). Activation of CXC chemokine receptor 2 (CXCR2) by chemokines (CXCL8 in humans; CXCL1 and CXCL2/3 in rats) also contributes to the increased release of EOPs from immune cells (Rittner et al., 2006a,b). Moreover, noradrenaline, stressful stimuli (e.g., experimental swim stress, surgery), and exogenous opioids have been shown to play a role in the release of leucocyte-derived opioid peptides (Binder et al., 2004; Machelska, 2007; Celik et al., 2016). Interestingly, one investigation demonstrated that peroxisome proliferator activated receptor-γ located in leukocytes, upon activation by 15d-PGJ2, releases β-endorphin and dynorphin into TMJ tissue (Macedo et al., 2016). More studies are needed to confirm the molecular modulation mechanism involved in opioid peptide release by orofacial inflamed/injured tissues.

**FIGURE 1:**
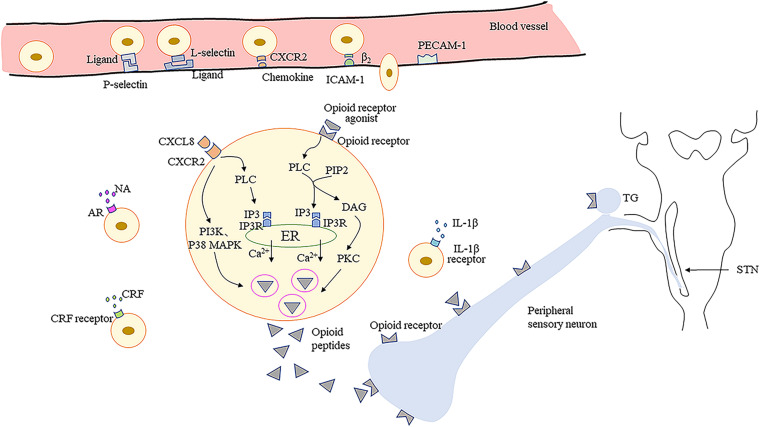
Migration of opioid peptide-containing immune cells and release of opioid peptides within peripheral inflamed or damaged tissues. Opioid peptide-containing immune cells migrate into inflamed or damaged tissues mediated by adhesion molecules. Release of opioid peptides is triggered by chemokines, inflammatory mediators or NA. Opioid peptide secretion is regulated predominantly by IP3R-triggered Ca^2+^ release from ER, partially by PI3K, and by p38 MAPK. Then opioid peptides bind to opioid receptors on peripheral terminals of sensory neurons resulting in pain inhibition. ICAM-1, intercellular adhesion molecule-1; PECAM-1, platelet-endothelial cell adhesion molecule-1; CRF, corticotropin-releasing factor; IL-1β, interleukin-1β; CXCR2, the CXC chemokine receptor 2; NA, noradrenaline; AR, adrenergic receptors; PLC, phospholipase C; PIP2, phosphatidylinositol-4,5-bisphosphate; IP3, inositol-3 phosphate; DAG, diacylglycerol; IP3R, inositol-3 phosphate receptors; ER, endoplasmic reticulum; PKC, phosphokinase C; PI3K, phosphoinositol-3-kinase; p38 MAPK, p38 mitogen-activated protein kinase; TG, trigeminal ganglion; STN, spinal trigeminal nucleus.

Furthermore, it has been demonstrated that EOPs are released from immune cells in a calcium-dependent manner (Rittner et al., 2006a). Activation of leucocyte opioid receptors by exogenous agonists promotes the activation of phospholipase C (PLC), which then hydrolyzes phosphatidylinositol-4,5-bisphosphate (PIP2) into inositol-3 phosphate (IP3) and diacylglycerol (DAG) (Celik et al., 2016). The binding of IP3 to IP3 receptors (IP3R) in the endoplasmic reticulum (ER) leads to mobilization of intracellular Ca2^+^, which then accelerates the secretion of EOPs (Celik et al., 2016). Phosphokinase C (PKC) activated by DAG may have a small effect on opioid peptide release (Feske, 2007). Evidence indicates that activation of CXCR2 by chemokines leads to opioid peptide secretion regulated predominantly by IP3R-triggered Ca^2+^ release from the ER, partially by phosphoinositol-3-kinase (PI3K), and by p38 mitogen-activated protein kinase (p38 MAPK) (Rittner et al., 2006a, 2007, 2009a,b). Then, the EOPs packaged in vesicular structures are translocated to the cell membrane (Mousa et al., 2004). Subsequently, EOPs bind to opioid receptors on the peripheral terminals of sensory neurons, resulting in pain inhibition (Figure 1).

#### Activation of PORs by Endogenous and Exogenous Opioids

Inflammatory conditions or nerve injuries increase the synthesis of opioid receptors in DRG neurons and enhance the peripherally directed axonal transport of opioid receptors (Sehgal et al., 2011; Stein and Machelska, 2011; Pettinger et al., 2013). Studies have revealed that bradykinin pretreatment induces a rapid and significant increase in the trafficking of DOR to the plasma membrane in cultured TG neurons (Patwardhan et al., 2005; Pettinger et al., 2013). PI3K expression has been demonstrated to promote the export of endogenous DOR in primary TG neurons (Shiwarski et al., 2017). Inflammation induces a decrease in extracellular pH, which enhances the interaction of PORs with G proteins and downstream signaling pathways (Vetter et al., 2006). Inflammation also induces the sprouting of opioid receptor-bearing peripheral nerve endings and damage to the perineural barrier, which facilitates access of opioid receptor agonists to PORs (Rittner et al., 2012). These factors enhance the analgesic effect of opioids in inflamed peripheral tissues. The underlying mechanism of neuropathic pain is relevant to inflammatory pain, as nerve injury is usually accompanied by inflammation. However, further research into ligand accessibility, affinity, coupling, and signaling of PORs in neuropathic pain conditions is still needed (Stein and Machelska, 2011).

**FIGURE 2:**
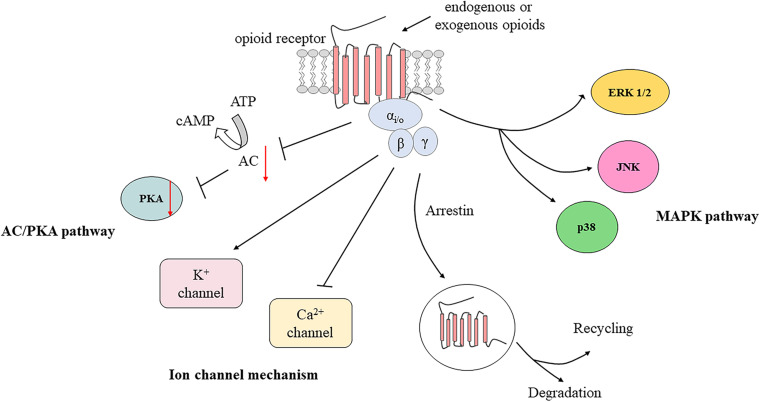
Activation of peripheral opioid receptors by endogenous and exogenous opioids. AC, adenylyl cyclase; cAMP, cyclic adenosine monophosphate; PKA, protein kinase A; ERK 1/2, extracellular signal regulated kinase 1 and 2; JNK, c-Jun N-terminal kinase; MAPK, mitogen-activated protein kinase.

After activation by endogenous or exogenous agonists, opioid receptors couple to heterotrimeric Gi/o proteins forming trimeric G protein complexes, which then dissociate into Gα and Gβγ subunits to interact with various downstream effectors. The Gα subunit inhibits adenylyl cyclase (AC) and cyclic adenosine monophosphate (cAMP) production (Brust et al., 2015). Hence, protein kinase A activity is suppressed due to decreased cAMP production, which causes the inhibition of numerous receptors or ion channels such as hyperpolarization-activated cyclic nucleotide-gated (HCN) channels, TRPV1, acid-sensing ion channels, and two pore domain channels (Francois and Scherrer, 2018). It has been reported that the application of protease-activated receptor-2 agonists induces antinociceptive effects of DOR by inhibiting cAMP accumulation in a capsaicin-evoked orofacial pain model (Patwardhan et al., 2006). Moreover, activation of opioid receptors can also inhibit voltage-gated calcium channel (VGCC) activity and open G protein-coupled inwardly rectifying K^+^ (GIRK) channels or ATP-dependent K^+^ (KATP) channels via the Gβγ subunit (Currie, 2010; Wang et al., 2010; Nockemann et al., 2013). GIRK and KATP channel subunits are expressed in TG neurons and are involved in orofacial muscle pain mediated by DOR (Niu et al., 2011; Saloman et al., 2011; Chung et al., 2014). Additionally, the MAPK pathway may also play a role in formalin-induced orofacial inflammatory pain (Huang et al., 2015; Kurose et al., 2017; Zhang et al., 2018). The L-arginine/NO/cGMP pathway also participates in opioid-mediated antinociception in response to orofacial pain (Napimoga et al., 2008; Clemente-Napimoga et al., 2009; Coura et al., 2017). In brief, activation of PORs leads to cellular hyperpolarization and suppresses excitability of peripheral sensory neurons, as well as to the decreased release of excitatory mediators, including substance P, calcitonin gene-related peptide (CGRP), and glutamate (Jin et al., 2006; Beaudry et al., 2011; Snyder et al., 2018; Figure 2).

Furthermore, exogenous opioid agonists injected close to peripheral nerves can produce analgesia by activating opioid receptors on neurons and immune cells (Plein and Rittner, 2018). Intriguingly, the activation of opioid receptors in leucocytes also induces the release of EOPs (Celik et al., 2016). Leucocyte-derived opioid peptides, in turn, induce analgesia by binding to opioid receptors on peripheral nerve sensory terminals. Therefore, the development of novel therapeutic strategies based on this neuron-immune cell analgesic pathway is important for orofacial pain treatment.

## Strategies for Orofacial Pain Control

Currently, medications for orofacial pain mainly include tricyclic antidepressants, serotonin–noradrenaline reuptake inhibitors, opioids, and non-steroidal anti-inflammatory drugs (Clark et al., 2016). However, these therapeutic agents are often ineffective in relieving pain and are associated with various adverse effects, such as respiratory depression, constipation, cardiotoxicity, and dizziness (Fornasari, 2017; Hossain et al., 2020). Undoubtedly, opioids are the most potent drugs for moderate-to-severe pain control (Wiffen et al., 2017), and drugs based on PORs are effective for pain relief without central side effects. Studies have reported that peripherally acting opioids significantly attenuate inflammatory and neuropathic pain (Tiwari et al., 2016, 2018; Bruce et al., 2019). Notably, peripherally acting opioids attenuate pain involving the dopaminergic and endocannabinoid systems, which require further intensive study (Zubrzycki et al., 2019; Vaidya et al., 2021). Interactions between immune cell-derived EOPs and PORs also provide novel insights into pharmaceutical development and alternative strategies for orofacial pain treatment. There are a range of approaches that can be used to enhance peripheral opioid analgesia based on EOPs and PORs in the orofacial region.

### Targeting EOPs in Immune Cells

It would be highly desirable to identify strategies for augmenting the production and release of opioid peptides in inflamed and damaged tissues. First, gene therapy is expected to an effective way to achieve long-term expression of EOPs. Administration of virus-based vectors expressing PENK into TG and whisker pad increases enkephalin expression and then produces analgesic effects in orofacial neuropathic and muscle pain models (Meunier et al., 2005; Tzabazis et al., 2014; Kramer et al., 2015; Ma et al., 2016; Meidahl et al., 2017). Common-employed vectors consist of plasmids, non-replicating adenoviruses, adeno-associated viruses, herpes simplex virus (HSV), and non-plasmid and non-viral DNA vectors (Machelska and Celik, 2018). Similarly, non-viral delivery of *Oprm1* into the cancer microenvironment produces endogenous analgesia through the secretion of β-endorphin in a preclinical oral cancer model (Yamano et al., 2017). Second, it is very important to enhance opioid production and release EOPs from immune cells into orofacial inflamed/injured tissue (Macedo et al., 2016; Scheff et al., 2019). Although inhibition of immune responses alleviates pain by decreasing inflammatory mediators at the beginning of inflammation or neuropathy, immunosuppressive interventions may exacerbate pain when peripheral inflammation and pain are already established (Machelska, 2011; Machelska and Celik, 2020). Therefore, it is significant to enhance the production and release of EOPs. It’s worth noting that anti-adhesion and anti-chemokine treatments may exacerbate pain due to the vital roles of adhesion molecules and chemokines in the migration of opioid-containing immune cells to injured tissue and the release of EOPs in inflamed tissue (Busch-Dienstfertig and Stein, 2010). Additionally, the application of enkephalinase inhibitors, such as neprilysin and aminopeptidase N, will effectively increase the duration and magnitude of analgesia by preventing released opioid peptide degradation (Carbone and Poole, 2020). How to translate these findings into clinical therapies for orofacial pain requires further investigation.

### Targeting PORs in Inflamed/Injured Tissue

The development of peripherally restricted opioid agonists has become a research hotspot for chronic pain treatment. Peripheral acting opioid receptor agonists have been reported to alleviate visceral pain and spinal nerve injury at pre-clinical level (Guan et al., 2008; Arendt-Nielsen et al., 2009). A mixed opioid DN-9 (Tyr-D.Ala-Gly-NMe.Phe-Gly-Pro-Gln-Arg-Phe-NH_2_) is developed to attenuate orofacial formalin pain via MOR and KOR (Zhang et al., 2018). Chemical modification of opioid receptor agonists is very important for exerting their effects on pain control. Low PH-dependent agonists or nanocarrier-based opioids favor receptor activation in the acid environments of inflamed and damaged tissues (Machelska and Celik, 2018). NFEPP [(±)-N-(3-fluoro-1-phenethylpiperidine-250 4-yl)-N-phenyl propionamide] is a fluorinated fentanyl derivative with maximal activity at low pH, which produces analgesia by activating PORs in rat paw inflammation and surgical incision models (Spahn et al., 2017). Furthermore, positive allosteric modulators potentiate receptor signaling by binding GPCRs at distinct sites to orthosteric ligands, such as endogenous and standard exogenous ligands (Machelska and Celik, 2018; Carbone et al., 2019). Augment the synthesis and transport of opioid receptors in peripheral tissues is also helpful in promoting analgesia. Although accumulating evidence exists regarding the functional roles of these approaches in pain modulation, little has been assessed in orofacial pain models.

## Conclusion

Inflammation or injury of peripheral nerves causes an increase in the expression of PORs on peripheral nerve terminals and production of immunocyte-derived EOPs. The activation of PORs by exogenous agonists or EOPs can produce analgesia in orofacial tissues without central side effects. However, investigations focused on peripheral opioid analgesia in orofacial pain are relatively fewer than those in trunk or limb pain. The precise mechanisms of analgesic effects induced by PORs and EOPs remain to be elucidated. Therefore, more animal and clinical studies are needed to identify the function and mechanisms involving PORs and EOPs in orofacial pain. The next challenges include developing peripherally restricted opioid agonists, accelerating the release of immune cell-derived EOPs to peripheral tissues, and augmenting the synthesis of opioid receptors on peripheral neurons. These findings may provide potential therapeutic strategies for the enhancement of analgesic efficacy in the treatment of orofacial pain.

## Author Contributions

QL designed and drafted the manuscript and figure. LM and SY analyzed the data. FH, WF, and HH revised the manuscript. All authors read and approved the final manuscript.

## Conflict of Interest

The authors declare that the research was conducted in the absence of any commercial or financial relationships that could be construed as a potential conflict of interest.
